# Host–Parasite Interactions Revisited: Evidence of Horizontal Transfer of a Transposable Element Between a Snail and Its Parasite

**DOI:** 10.1093/gbe/evag107

**Published:** 2026-05-08

**Authors:** Toby Brann, Fernanda Souza de Oliveira, Andrés Iriarte, Anna Victoria Protasio

**Affiliations:** Department of Pathology, University of Cambridge, Cambridge CB1 2PQ, UK; Department of Pathology, University of Cambridge, Cambridge CB1 2PQ, UK; Programa de Pós-Graduação Em Genética, Universidade Federal Do Paraná, Centro Politécnico, Avenida Coronel Francisco H. Dos Santos, 100, Curitiba, Paraná 81531-990, Brazil; Departamento de Biotecnología, Instituto de Higiene, Universidad de la República, Montevideo 11600, Uruguay; Department of Pathology, University of Cambridge, Cambridge CB1 2PQ, UK; Christ’s College, University of Cambridge, Cambridge CB2 3BU, UK

**Keywords:** *Schistosoma mansoni*, transposable element, horizontal gene transfer, host–parasite relationship, Schistosomatidae

## Abstract

In eukaryotes, horizontal gene transfer (HGT) often involves transposable elements (TEs), host–parasite relationships, aquatic environments, or any of them combined. Horizontal transfer of transposable elements is both impactful, owing to the subsequent transposition burst, and insightful, providing information on organisms’ evolutionary history. The flatworm *Schistosoma mansoni* is a human parasite with two free-living aquatic stages (intercalated between a definitive human host and intermediate snail host) and has a sizable TE content. We aimed to identify and characterize potential instances of HGT leveraging new genomic resources available. Using the latest chromosome-scale genome assembly and available TE sequences for the *S. mansoni* genome, we identify that two TEs, named Perere-3 and Sr3, are putatively horizontally transferred. We demonstrate the presence of these TEs in the genomes of *Schistosoma* spp. intermediate hosts, most likely explained by HGT. Perere-3/Sr3 were also found across a wide range of additional organisms not susceptible to schistosome infection, including turtles, fish, and other mollusks. We propose that the patchy distribution of Perere-3/Sr3 across the phylogenetic tree is best explained by HGT. Our synonymous substitution calculations further support HGT, as divergence between schistosome and snail TE sequences is markedly lower than that observed for conserved orthologous genes. We propose that HGT is likely linked to schistosomes’ parasitic nature as several snail species sharing the elements are susceptible to infection. However, the rationale for the presence of Perere-3/Sr3 in species beyond this relationship is unknown.

Significance Horizontal transfer of DNA between animals is thought to be rare, and many previous claims, especially in parasites, have been questioned due to contamination or limited genomic data. Using high-quality genome assemblies, we show that two mobile DNA elements are shared between the human parasite *Schistosoma mansoni* and its snail intermediate hosts, despite these species being distantly related. This finding supports the hypothesis that intimate host–parasite interactions can facilitate the movement of genetic material across species boundaries, offering new insight into how parasitism can shape genome evolution.

## Background

Horizontal gene transfer (HGT) refers to DNA acquisition through mechanisms other than reproduction. Although examples are found in all realms, HGT in eukaryotes occurs at a rate approximately 80 times less than in prokaryotes (Van Etten and Bhattacharya 2020). This may be partly due to additional physical barriers present in eukaryotes, such as the nuclear envelope, compartmentalization of the germline where the transferred genes must be present to be propagated to future generations, and, in many metazoans, a protective skin or dermis (Kurland et al. 2003; Keeling 2024).

Host–parasite relationships intrinsically reduce physical separation between organisms and are commonly described in instances of HGT (Gilbert et al. 2010; Wijayawardena et al. 2013; Yang et al. 2016). Metazoan parasites live for varied, but often extended, periods of time in direct contact with their hosts, often feeding on host tissue. This intimate host–parasite relationship provides an excellent environment for exposure to the other organism's DNA. In addition to parasitism, HGT has historically been associated with aquatic environments, where shared exposure to the same water matrix was hypothesized to facilitate genetic exchange between organisms (Abe et al. 2020; Pérez-Etayo et al. 2020). However, more recent evidence suggests that HGT may not occur at higher rates in aquatic systems compared with terrestrial environments (Muller et al. 2025).

Transposable elements (TEs) are genetic entities capable of mobilization within genomes. They are more frequently implicated in horizontal transfer events (referred to as “horizontal transfer of TEs” or HTT) than “traditional” (ie non-mobile) genes due to their inherent capacity to encode enzymes that mediate their excision and insertion into genomes and, excluding DNA transposons, increase in copy number increasing the probability of retention in successive generations (Schaack et al. 2010; Gilbert and Feschotte 2018). TEs are often present in many copies and can occupy large fractions of host genomes, such as between 52% and 69% in Humans (de Koning et al. 2011; Tang et al. 2018) and 78% in Antarctic krill (Shao et al. 2023).

TE mobilization has consequences in all genomes, disrupting or altering host genes and providing a substrate for exaptation or structural rearrangements (Young et al. 2020). To mitigate the potential disruptive effects of TEs, organisms have evolved TE suppression mechanisms, including posttranscriptional silencing that degrades nascent TE mRNAs and heterochromatin formation of TE-derived DNA which prevents TE transcription (Slotkin and Martienssen 2007). Foreign TEs acquired by HTT are more likely to escape any inherent host resistance depending on the relationship with previously invaded TEs, resulting in a “transposition burst”: a rapid increase in TE copy number in the genome. Such a burst can change genome size, interrupting and shuffling genes and skewing GC content. For example, the horizontally transferred LINE-RTE BovB constitutes 18% of the cow (*Bos taurus*) genome and is also present in other mammals such as the platypus (*Ornithorhynchus anatinus*) and multiple lineages of snake (Walsh et al. 2013; Puinongpo et al. 2020). BovB dissemination was likely mediated by parasitic ticks with a broader range of ancestral hosts than currently observed (Ivancevic et al. 2013). Examples such as this offer valuable insights into the evolutionary relationships and natural history of implicated species.

With approximately 40% of its genome derived from TEs, *Schistosoma mansoni*, a human parasitic flatworm and the best characterized species of the Schistosomatidae family, is considered TE-rich (International Helminth Genome Consortium 2019). These parasites have complex life cycles alternating between asexual reproduction in snails and sexual reproduction in vertebrate hosts (such as humans) with two short-lived aquatic stages: miracidia and cercariae (Nelwan 2019). Adult schistosomes can survive in their vertebrate hosts for many years or decades, the oldest recorded survival of individual worms being 37 years (Chabasse et al. 1985). Parasite persistence in the snail host is variable, but lifecycle completion requires at least 5 weeks in laboratory conditions (Kapp et al. 2003).

The theoretical basis of *S. mansoni* involvement in HTT seems plausible given that the parasite has multiple aquatic life stages, extended periods of host association, and at least one third of its genome is TE-derived (International Helminth Genome Consortium 2019), characteristics known to promote HTT (Gilbert et al. 2010; Abe et al. 2020; Pérez-Etayo et al. 2020). In this context, others have previously searched for HTT events in schistosomes. For example, an HTT event from salmonid to schistosome was proposed based on analysis of an EST library and subsequent amplification of salmonid-specific transposon-like sequences in *Schistosoma* DNA/cDNA (Melamed et al. 2004). This work was later disproved on the basis that the level of salmon DNA contamination in the EST library was much higher than expected, potentially due to salmon sperm DNA being used as a DNA carrier during DNA isolation (Grunau and Boissier 2010; Wijayawardena et al. 2015). Other claims of HTT in *Schistosoma* including transfers of major histocompatibility complex sequences from mice or rabbits (Imase et al. 2001; Zhao et al. 2009), DNA transposons from fish and frogs (Leaver 2001), receptor sequences from mammals (Hu et al. 2003), and albumin from mice (Williams et al. 2006; DeMarco et al. 2007) have equally been contested based on DNA contamination, inadequate sampling of taxa, sequence conservation, or incomplete/fragmented genome assemblies (Wijayawardena et al. 2015). More recently, the transfer of a functional *cki* homolog in parasitic flatworms (including *S. mansoni*) from an unidentified metazoan has been proposed (Wendt and Collins III 2024), perhaps representing one of the few (as yet) undisputed cases of HGT.

Recently, a chromosomal-level genome assembly for *S. mansoni* (Buddenborg et al. 2021) and high-quality genomes of *Schistosoma* snail hosts (Nong et al. 2022; Young et al. 2022; Bu et al. 2023) have been made available, opening the opportunity for a reliable, comprehensive investigation into horizontal transfer in a medically relevant organism with a high potential for gene transfer. Here, we leverage these genomic resources to identify and characterize instances of HTT between *S. mansoni* and its hosts and wider metazoans implicated in this phenomenon.

## Results

### Finding *Schistosoma* TE-like Sequences in the Parasite's Intermediate Hosts

**Fig. 1.**
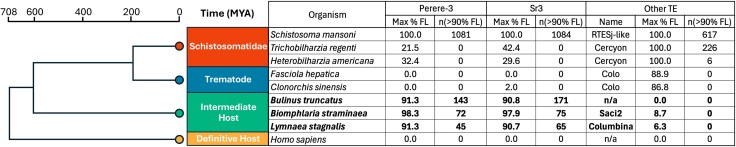
The genome of the intermediate host of *S. mansoni* contains *S. mansoni*-like TEs. Selected organisms with varying relationships to *S. mansoni* were scanned with a relaxed BLASTN to identify the presence of *S. mansoni* TEs in these genomes. “Max % FL” represents the highest percentage of the *S. mansoni* TE consensus sequence that was mapped to the genome. “n(90% FL)” was calculated as the number of copies identified over 90% of the full length of the respective elements. The highest non-Perere-3 or non-Sr3 element (“Other TE”) is also shown to highlight potential differences between Perere-3/Sr3-derived results and other elements. Simplified phylogeny (left) was extracted from timetree.org (Kumar et al. 2022), representative genomes used for each group highlighted with *.

We explored the possibility of HTT in *S. mansoni* using a relaxed BLASTN search of known *S. mansoni* TEs against other Schistosomatidae (*Trichobilharzia regenti*, *Heterobilharzia americana*) and trematodes (*Fasciola hepatica*, *Clonorchis sinensis*) as well as intermediate (*Bulinus truncatus*, *Biomphalaria straminea*, *Lymnaea stagnalis*) or definitive hosts (*Homo sapiens*). Using this approach, we found significant similarity to two LINE-RTEs known as Perere-3 and Sr3 in the three genomes corresponding to *Schistosoma* intermediate hosts, ie mollusks (Fig. 1). Prospective TEs are present at near full length of the *S. mansoni* consensus sequence used as query in the search (91.3% and 90.8% in *Bu. truncatus*, 98.3% and 97.9% in *Bi. straminea*, and 91.3% and 90.8% in *L. stagnalis* for Perere-3 and Sr3, respectively), suggesting similarity is not simply due to the presence of highly conserved TE domains but instead represents a full-length match. Additionally, in these organisms, many near-full-length (more than 90% full length, approximately 2,900 bp for Perere-3 and Sr3) copies were identified, indicating transposition of these elements. In contrast, few “Other TEs” were identified above the e-value filter in intermediate snail hosts. We did not detect significant hits of Perere-3/Sr3 in the other trematodes closely related to *Schistosoma*, namely, *C. sinensis* or *F. hepatica*. As molluskan intermediate hosts are evolutionarily more distant from *S. mansoni* than the trematodes, the presence of Perere-3 and Sr3 in the snails is unlikely to be a product of vertical inheritance. This first observation led us to hypothesize that an HTT event between mollusks and their parasites could have occurred, either directly or indirectly.

### Perere-3 and Sr3 in the *S. mansoni* Genome

**Fig. 2.**
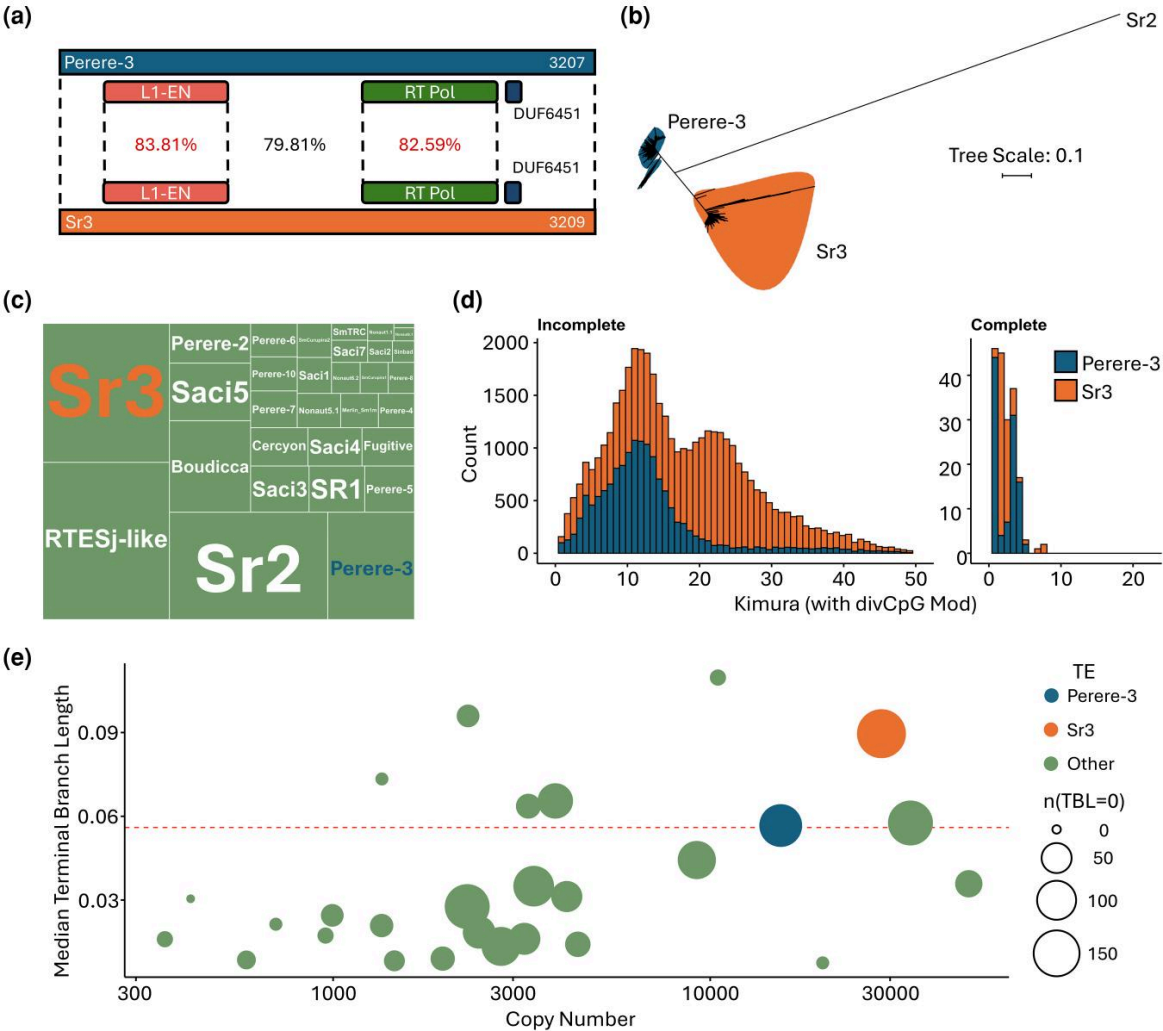
Perere-3 and Sr3 are similar LINE-RTEs with different transposition histories within the *S. mansoni* genome. a) Protein domain topology of Perere-3 and Sr3, with percentage identity (DNA) of the entire element and the L1-endonuclease (L1-EN) and reverse transcriptase polymerase (RT Pol) protein domains. b) Tree of full-length (>2,900 bp) TE insertions in the *S. mansoni* genome with Sr2 LINE-RTE as outgroup. c) Relative genomic coverage of TEs of the *S. mansoni* genome. d) Kimura profile of Perere-3 and Sr3 in the *S. mansoni* genome, divided into “incomplete” and “complete” elements, where complete implies at least 99% of both the L1-EN and RT Pol domains. e) “Terminal branch length” (TBL) analysis of all *S. mansoni* TE copies, assessing copy number against median terminal branch length. The size of the datapoint indicates the number of instances where the TBL was 0. Red dashed line represents the mean terminal branch length of all elements.

We characterized Perere-3 and Sr3 in the *S. mansoni* genome. In the first instance, we took the sequences of Perere-3 and Sr3 previously described by Venancio et al. (2010) and used the most recent version of the *S. mansoni* assembly to update their consensus sequences using a manual curation approach that better represents the range of TE insertions found in the genome. The resulting consensus sequences for Perere-3 and Sr3 are 3,207 and 3,209 bp, respectively (File S2). With 79.8% identity between them, these elements have high sequence similarity and fall short (by 0.2%) of being considered elements of the same TE family (Wicker et al 2007). Both elements encode full-length L1-endonuclease (L1-EN) and the reverse transcriptase (RT Pol), for which their amino acid percentage identities are 83.81% and 82.59% related to one another, respectively (Fig. 2a).

Despite their close sequence similarity, these TEs are considered to transpose independently from one another. To confirm this, we retrieved from the genome all Perere-3 and Sr3 copies with a minimum length of 3,200 bp and built a sequence similarity dendrogram (Fig. 2b). Our results show that sequences arising from Perere-3 or Sr3 TE transposition events segregate into two distinct clusters. Furthermore, the relative distance between these two LINE-RTEs and others from the same organism suggests that Perere-3 and Sr3 may have relatively recently shared a common TE ancestor (Fig. 2b). Differential transposition activity has resulted in these elements having different proportions of genomic coverage: Perere-3 and Sr3 occupy 2.1% and 4.0% of the genome, respectively (Fig. 2c). Further evidence for their independent activity can be seen in their profile of accumulated mutations represented in the differential kimura distributions (Fig. 2d). Kimura distances represent the evolutionary distance of a given insertion from the consensus sequence from which relative age may be inferred. Our data show that Sr3 TEs have a distinct bimodal distribution of kimura values, with a peak at both 12 and 22, in comparison to Perere-3 which is unimodal with a single peak corresponding to a kimura value of 12. This indicates differential bursts of transposition between these elements and pointing to independent transposition.

To further understand the dynamics of Perere-3 and Sr3 in *S. mansoni*, particularly given their potential involvement in horizontal transfer events, we aimed to assess ongoing transposition activity of these elements. Based on their completeness, these elements can transpose: of the 43,800 Perere-3/Sr3 annotated fragments, 432 (approximately 1%) have at least 99% of the length of the protein domains required for TE transposition, namely, the L1-endonuclease (L1-EN) and the reverse transcriptase (RT pol) domains. However, final proof of ongoing or very recent transposition would be given by the presence of identical TE insertions. To this end, we use a Terminal Branch Length or TBL approach which provides an estimation of the relative age of all TE copies (Chang et al. 2022), with low TBLs values indicating more recent transpositions. We demonstrate that Sr3 and Perere-3 have 173 and 124 copies with a TBL of 0 (Fig. 2e), indicating many identical pairwise matches and suggesting that these transposition events are recent, with insufficient time to accumulate mutations.

### Evolutionary Spread and Activity of Perere-3/Sr3 Across *Schistosoma* and Molluskan Hosts

In the first section, we showed that Perere-3 and Sr3 are found extensively across the *Schistosoma* genus but not in full length nor in high numbers in the wider Trematoda class (Fig. 1). We broadened our search beyond the *S. mansoni* genome to evaluate the presence and extent of transposition across the *Schistosoma* genus and to more accurately determine the evolutionary timing of a potential transfer event. We searched for Perere-3 and Sr3 sequences (independently) in 11 additional *Schistosoma* genomes and found that all contained full copies of the TEs (Table 1). The results returned for Perere-3 and Sr3 were highly similar, arising from the underlying percentage identity of the initial TE consensus sequences (79.8%), falling just outside of the definition of being classified as a single element. Therefore, when extending our search of these sequences outside of Schistosomatidae, the two sequences were considered functionally equivalent, as “Perere-3/Sr3.”

**Table 1:** Full-length copies of Perere-3 and Sr3 are found across *Schistosoma* spp. Perere-3 and Sr3 copies identified using the “relaxed BLASTN” and manually curated *S. mansoni* Perere-3 and Sr3 consensus sequences

Species	Perere-3% genome	Perere-3 *n* (>2,900 bp)	Perere-3 max full length	Sr3% genome	Sr3 *n* (>2,900 bp)	Sr3 max full length
*S. japonicum*	3.81	977	3,239	3.88	982	3,242
*S. turkestanicum*	5.98	1,156	3,225	5.36	1,135	3,241
*S. rodhaini*	5.31	1,563	3,241	5.81	1,574	3,241
*S. mansoni*	4.91	1,073	3,241	5.43	1,079	3,244
*S. spindale*	5.5	1,482	3,237	6.04	1,486	3,252
*S. margrebowiei*	4.8	891	3,257	5.35	903	3,257
*S. mattheei*	5.1	1,277	3,247	5.65	1,284	3,287
*S. intercalatum*	5.13	1,253	3,256	5.69	1,257	3,256
*S. haematobium*	4.81	1,511	3,253	5.32	1,514	3,269
*S. guineensis*	4.94	1,130	3,253	5.49	1,134	3,268
*S. curassoni*	4.96	1,168	3,259	5.51	1,177	3,266
*S. bovis*	4.93	1,115	3,249	5.46	1,122	3,249

All genomes used are available on WormBase ParaSite, with accessions listed in Table 4.

**Fig. 3.**
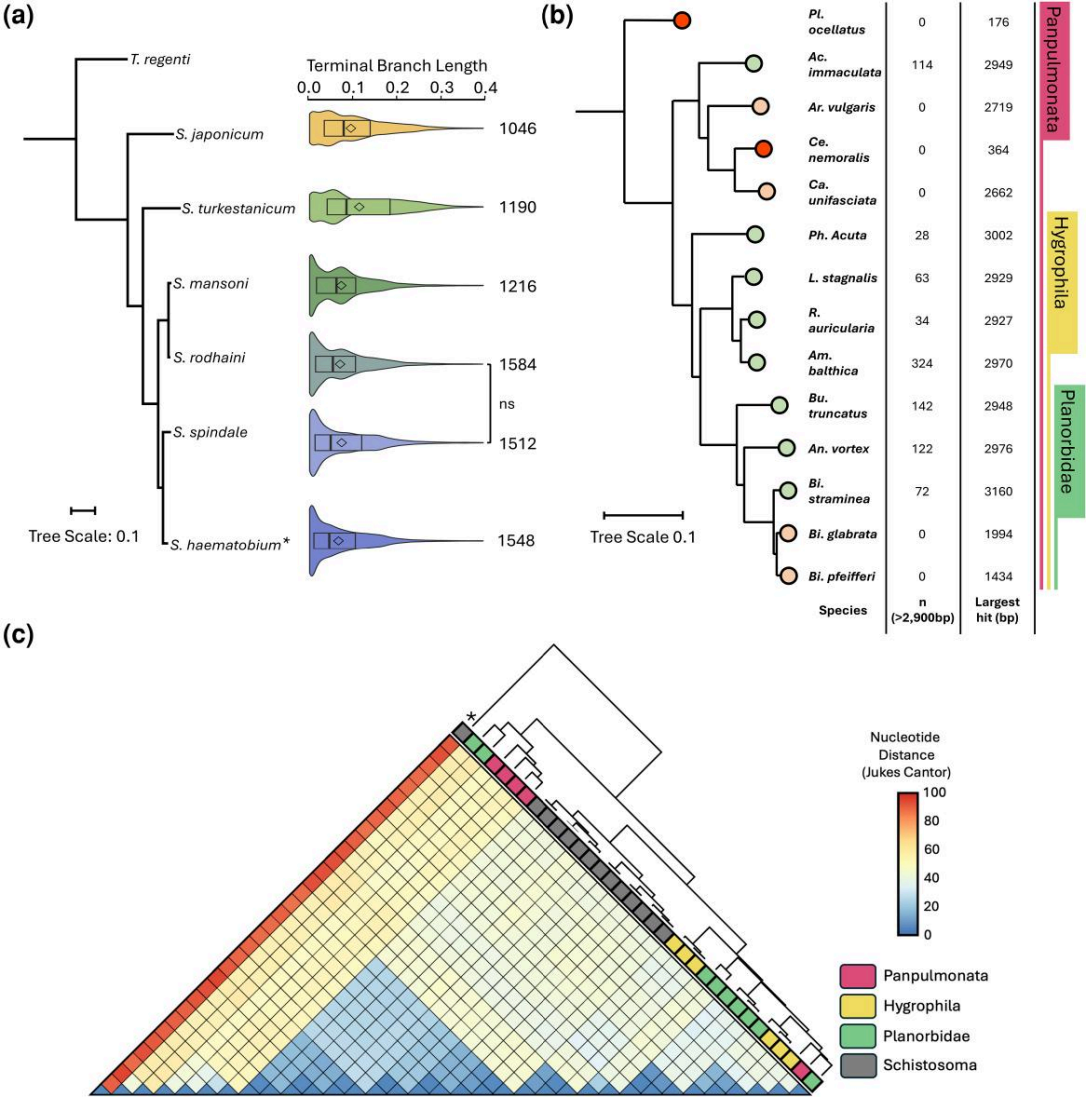
*S. mansoni* Perere-3 and Sr3-like sequences are found across *Schistosoma sp.* and mollusks, including snails susceptible to infection. a) *Schistosoma* species tree and terminal branch length (TBL) distribution of Perere-3 and Sr3-like insertions aligned to the said tree. The species tree was constructed from reciprocal orthologs across all species. *Schistosoma* phylogeny was constructed with all species available in WormBase ParaSite. *S. mattheei*, *S. intercalatum*, *S. haematobium*, *S. guineensis*, *S. curassoni*, and *S. bovis* were collapsed into a single clade representing the *S. haematobium* group (*), in line with previous phylogenetic assessment of *Schistosoma* (Leger and Webster 2017). *Trichobilharzia regenti* was used to root the tree, although no full-length Perere-3/Sr3-like sequences were identified in this organism (Fig. 1). Diamonds indicate the mean. Testing for TBL differences between species with a Kruskal–Wallis test and Dunn's post hoc analysis demonstrated significance (*P*  *<*  *0.001*) across all pairwise-species comparisons except *S. spindale* and *S. rodhaini* (*P*  *>*  *0.05*). Number of elements greater than 2,900 bp is shown to the right of the violin plot. b) Rooted species tree of Panpulmonata, including Planorbidae snails, the intermediate hosts of *Schistosoma* spp. and related *Hygrophila* snails. *Plakobranchus ocellatus* was used as an outgroup. Molluskan genes were obtained with BUSCO (see Methods). Leaves are color-coded to indicate presence of elements over 2,900 bp (green), over 1,000 bp (orange) or shorter (red). c) Heatmap representing distance matrix (DNA) of manually curated TE consensus sequences from *Schistosoma* and panpulmonate snails alongside hierarchical clustering tree. Snails are labeled Planorbidae (green), *Hygrophila* (yellow) or panpulmonate (pink). *S. mansoni* LINE-RTE Sr2 was added as an outgroup, as indicated by the “*” in the tree.

Our analysis of TBL across the *Schistosoma* genus (Fig. 3a) suggests that these elements have been active throughout speciation. While similar numbers of near-full-length (>2,900 bp) elements are observed across *Schistosoma*, Perere-3 and Sr3 from *S. japonicum* and *S. turkestanicum* have higher TBLs medians and fewer near 0 TBLs, indicating that a higher proportion of transposition events in these organisms are older than in those species most recently diverged (Fig. 3a). We found a significant increase in TBL between *S. japonicum* and *S. turkestanicum* and the newer diverged schistosomes (Kruskal–Wallis and Dunn's test, *P*  *<*  *0.001*). Furthermore, we found syntenic copies of Perere-3/Sr3 in reciprolog genes in *Schistosoma* species spanning the *S. mansoni* and *S. haematobium* clades (Fig. S1), demonstrating that some insertions have been vertically inherited. Together with the widespread presence and activity of these elements across *Schistosoma* species, evidence suggests that the horizontal transfer event likely occurred in a common ancestor of the genus.

Having found the extended presence of Perere-3 and Sr3 in a wider range of *Schistosoma* spp., we wanted to test whether these TEs are also present in the respective intermediate hosts (family: Planorbidae) and other mollusks across Panpulmonata. We used the “relaxed BLASTN” approach with *S. mansoni* Perere-3 and Sr3 as queries on the genomes of selected Planorbidae and Panpulmonata and built a corresponding species tree to represent species relatedness (Fig. 3b). We found significant BLASTN hits corresponding to more than 90% of the full length of *S. mansoni* Perere-3 and Sr3 consensus sequences (green in Fig. 3b, Table S1) in eight molluskan species. For instance, *Ampullaceana balthica* contains 324 copies over 2,900 bp in length (ie full length). Two of the three species of *Biomphalaria* (viz. *Bi. glabrata* and *Bi. pfeifferi*) have a marked reduction in the number of copies as well as their maximum length, suggesting TEs in these species are no longer active, and the remaining identifiable copies have accumulated many mutations.

To explore the relationship between *Schistosoma* and molluskan Perere-3/Sr3-like sequences, we manually curated these TEs from the 12 molluskan genomes for which significant BLASTN hits were found. To find out how closely related these sequences are to each other, we built a nucleotide distance matrix (Fig. 3c) of the *Schistosoma* and molluskan TEs consensus sequences. A hierarchical clustering approach was used to identify intersequence relationships, and we found that *Schistosoma* Perere-3/Sr3-like sequences cluster within the curated molluskan TEs (gray, Fig. 3c). The high similarity observed between *Schistosoma* and molluskan Perere-3/Sr3-like elements and positioning of the *Schistosoma* clade within a continuum of molluskan TE sequences further adds to the evidence of a potential horizontal transfer event. Additionally, we found Perere-3/Sr3 in *Hygrophila* snails, which are not Planorbidae and are not known to be susceptible to *Schistosoma* infection.

### Synonymous Divergence (dS) Analysis

To further investigate the relationship between *Schistosoma* and molluskan Perere-3/Sr3 instances, we analyzed the distribution of synonymous substitutions (dS) among the well-conserved reverse transcriptase polymerase (RT pol) domains of Perere-3/Sr3 sequences. We hypothesized that if these TEs were the product of horizontal transfer, their dS values would be lower than those observed for orthologous host protein-coding genes. To approximate the level of neutral divergence accumulated since the diversification of the genus *Schistosoma*, we calculated dS between RT pol sequences from *S. mansoni* and *S. japonicum*. We then estimated dS between RT pol sequences from schistosomes and snail species to represent divergence predating the split between flatworms and mollusks. As a reference for vertically inherited genes, we calculated synonymous divergence among BUSCO orthologs across the same species set.

We found that the synonymous divergence of RT pol sequences between *S. japonicum* and *S. mansoni* ranged from 0.41 to 0.70, with a mean of 0.59 (SD ± 0.08) and a median of 0.63. In contrast, RT pol sequences between schistosome and snail species showed substantially higher divergence, with dS values ranging from 1.11 to 1.43 (mean 1.27, SD ± 0.08; median 1.31). For comparison, BUSCO orthologs between schistosome and snail species showed dS values ranging from 2.32 to 39.02, with a mean of 9.19 (SD ± 5.08) and a median of 8.03. These values suggest that synonymous divergence among many orthologous genes is saturated at this evolutionary depth, whereas the RT pol sequences fall well below this saturation range. Among schistosome species, BUSCO orthologs showed dS values ranging from 0.29 to 1.80 (mean 0.74, SD ± 0.21), indicating that vertically inherited genes accumulate synonymous substitutions at rates comparable to those observed for RT pol sequences within the genus (Fig. S2 and Table S2).

Overall, these results show that synonymous divergence among RT pol sequences between schistosome species is comparable to that observed among vertically inherited orthologous genes, whereas divergence between schistosomes and snails remains substantially lower than that observed for conserved orthologs across these phyla. Together with the continuous distribution of Perere-3/Sr3 elements in *Schistosoma* and their patchy distribution in snails, these results suggest that the most likely explanation for the origin of Perere-3/Sr3 in schistosomes is horizontal gene transfer between these two groups.

### Assessment of Perere-3 and Sr3 Across Metazoa

**Fig. 4.**
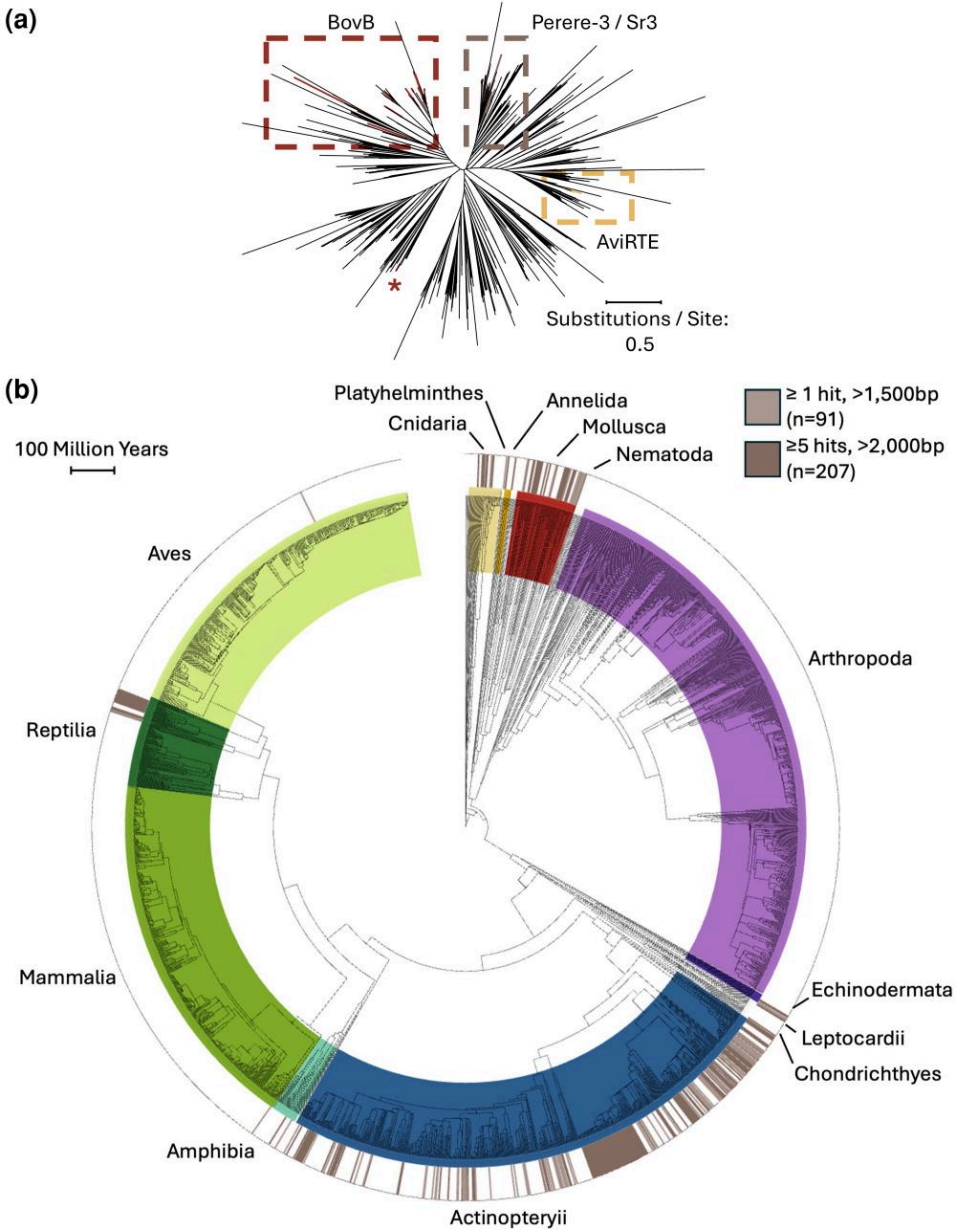
Perere-3 and Sr3 are found across Metazoa. a) Dendrogram of consensus sequences of LINE-RTEs found in RepBase plus *Schistosoma* and mollusk Perere-3/Sr3-like sequences, highlighting known horizontally transferred groups BovB (Ivancevic et al. 2013) and AviRTE (Suh et al. 2016). * indicates additional BovB sequence that did not cluster within other BovB sequences. b) Presence of Perere-3/Sr3-like sequences across metazoan genomes available at Timetree.org (Kumar et al. 2022). Relevant hits were classified as either weak (light brown: at least one hit > 1,500 bp) or strong (dark brown: at least one hit > 2,000 bp and at least five hits > 1,000 bp). Perere-3 and Sr3 were used as a query, results shown for the TE generating higher results, TEs were plotted separately in Fig. S2.

To assess the relationship between *Schistosoma*/molluskan Perere-3 and Sr3-like sequences and the wider LINE-RTE superfamily of TEs, we extracted consensus sequences of LINE-RTEs from the RepBase database (Bao et al. 2015) and used these to construct a sequence similarity dendrogram. Our analysis included two well-known horizontal TE transfers of LINE-RTEs, namely, BovB (Ivancevic et al. 2013) and AviRTE (Suh et al. 2016). We show that Perere-3 and Sr3 sequences from *Schistosoma* and Gastropoda cluster tightly together and, at the same time, separately from the known TE transfers, indicating a distinct transfer event from those previously described (Fig. 4a).

Notably, several LINE-RTEs from RepBase clustered around our curated Sr3/Perere-3 sequences, such as those from *Crassostrea gigas* (Pacific oyster), *Aplysia californica* (Californian sea hare, a sea slug), *Saccoglossus kowalevskii* (acorn worm, a free-living, hemichordate), *Danio rerio* (zebrafish), and *Chrysemys picta bellii* (Western painted turtle). Given this clustering, we hypothesized that Perere-3/Sr3 may exist outside *Schistosoma* and mollusks, and, as RepBase is limited to user-submitted, previously curated TEs, we extended our search to all available, high-quality metazoan genomes. This was done with the relaxed BLASTN approach using *S. mansoni* Perere-3 and Sr3 as queries against all metazoan genomes (scaffold N50 > 1Mbp) available in GenBank (*n* = 3,446, Table S3). When assessing the potential presence of Perere-3/Sr3 in metazoan genomes, an organism was considered a “weak” candidate if it returned a single blast hit >1,500 bp, while a “strong” candidate required at least one larger than 2,000 bp and at least five hits >1,000 bp. Stringency around the number of hits identified helps to differentiate between potential contamination resulting in a single hit (“weak” candidates) and bona fide hits, which would have many copies (“strong” candidates). Using these criteria, we found that out of the 3,446 genomes tested, 117 organisms were “weak” candidates for Perere-3/Sr3 HTT while 256 of these were “strong” candidates. To visualize the presence/absence of Perere-3/Sr3-like sequences in these species and their respective relationships, we used timetree.org and filtered for genomes for which speciation data were available in this database (*n* = 2,271). From this subset of genomes, 91 and 207 were “weak” and “strong” candidates for Perere-3/Sr3 presence, respectively.

**Fig. 5.**
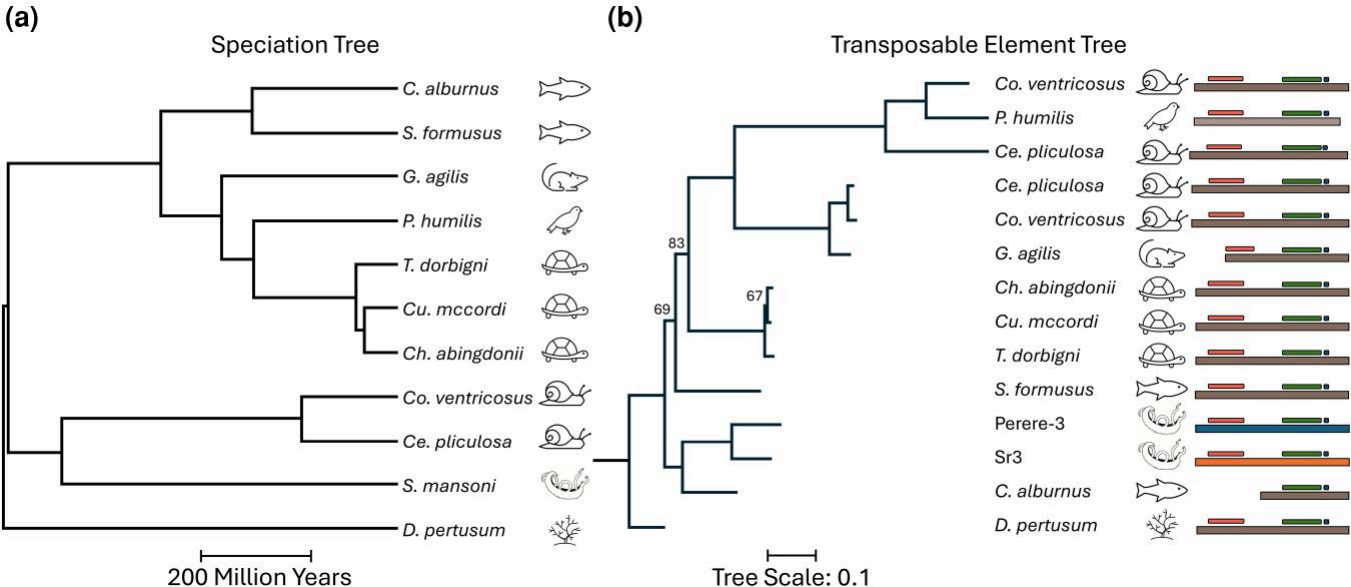
Juxtaposition of TE and species trees highlights the phylogenetic incongruence between them and is evidence in support of the horizontal transfer of Sr3 and Perere-3 a) speciation tree generated with timetree.org showing divergence times of selected organisms. b) Tree of manually curated TE DNA consensus sequences of Perere-3/Sr3 from the same organisms in A. Two distinct elements were curated for *Conus ventricosus* and *Cerithideopsis pliculosa*. *Culter alburnus* and *Gracilinanus agilis* curated elements were 5′ truncated. Ultrafast bootstrap values lower than 98 are shown. TE domains of curated elements are represented with boxes to the right of the panel; L1-EN (red), RT_Pol (green) and DUF-6451. Protein domains of all consensus sequences were mapped using the methodology for Fig. 2a.

We found Perere-3/Sr3-like sequences discontinuously across the metazoan tree (Fig. 4b), including reptiles, such as the previously mentioned *C. p. bellii*, amphibians, marine invertebrates, mollusks, Cnidaria, a single bird and a single mammal, and more extensively across fish. Such a diverse number of taxa from which these hits derived may suggest that Perere-3/Sr3 have been extensively transferred across metazoans or independently degenerated. In line with our approach to consider Perere-3 and Sr3 together in nonmetazoan organisms due to their percentage identity, highly similar results were seen for each of Perere-3 and Sr3 when searched against metazoan genomes (Fig. S1). Of the 298 organisms identified as containing Perere-3/Sr3 sequences, we highlight several species (Table 2) which were selected either due to having large numbers of hits over 1,000 bp, having the highest single largest hits, or being notable taxonomic clades. To validate the presence of Perere-3/Sr3 in these genomes and investigate TEs and species relatedness, we curated the identified Perere-3/Sr3-like sequences in these species. We first evaluated their sequence similarity and presence of conserved protein domains and found that, despite a large evolutionary distance across taxa (Fig. 5a), all but two species of curated TEs encode full-length L1-end and RT pol domains and shared overall TE domain topology (Fig. 5b, Fig. S3). We then compared a dendrogram of curated consensus sequences to the corresponding species tree, demonstrating extensive phylogenetic incongruence between the species and TE trees (Fig. 5). For example, despite fish and *S. mansoni* diverging more than 600 million years ago (Fig. 5a), curated Perere-3/Sr3-like sequences from fish are closely related to their *S. mansoni* counterparts, contradictory to what would be expected by vertical inheritance. Such phylogenetic incongruence among species, despite high sequence similarity, is consistent with horizontal transfer of Perere-3 and Sr3 beyond *Schistosoma* and mollusks, potentially extending across Metazoa.

**Table 2:** Select metazoan species identified from Fig. 4

Taxa	Organism	Synonym	Max length	% ID	*n* (>1,000 bp)
*Schistosoma*	*S. japonicum*	-	3,239	74.1	5,349
Tortoise/turtle	*Chelonoidis abingdonii*	Pinta Island tortoise	3,199	66.2	78
	*Trachemys dorbigni*	D’Orbigny’s slider	3,195	65.6	104
	*Cuora mccordi*	McCord's box turtle	3,189	65.9	692
Fish	*Scleropages formosus*	Asian arowana	3,192	65.3	62
	*Sinocyclocheilus grahami*	Golden-line barbel	3,036	72.1	4
	*C. alburnus*	Topmouth culter	3,042	72	7
	*Megalobrama amblycephala*	Wuchang bream	3,037	71.7	2
Mollusk	*C. pliculosa*	Plicate horn shell	3,039	68.1	2019
	*C. ventricosus*	Mediterranean cone	3,172	66.6	800
Coral	*Desmophyllum pertusum*	*Lophelia pertusa*	3,028	68.9	55
Mammal	*G. agilis*	Agile gracile opossum	1,619	60.4	28
Bird	*P. humilis*	Grount tit	2,582	64.6	1

Species were chosen based on the presence of Perere-3/Sr3 with regards to highest maximum length, highest percentage identities, and highest *n* (>1,000 bp) found. In addition to these, notable taxa identified were also included (coral, mammal, and bird).

## Discussion

Our preliminary investigation using BLASTN on the genomes of trematodes and *Schistosoma* molluskan hosts (viz. human and Planorbidae snails) with *S. mansoni* TEs, revealed that two elements, Perere-3 and Sr3, are well represented in the genomes of *Schistosoma* spp.'s intermediate hosts (*L. stagnalis*, *Bu. truncatus*, *Bi. straminea*), while no other TE shares the same degree of similarity among members of this group. Perere-3 and Sr3 are absent from the definitive human host and from the non-*Schistosoma* trematode genomes sampled, suggesting either loss in the trematode lineages sampled or a later gain in *Schistosoma*. Owing to the parasitic relationship characterized by prolonged physical contact between worm and snail, we hypothesized that Perere-3 and Sr3 could represent examples of horizontally transferred TEs. Previously documented horizontal transfer events involving *S. mansoni*, eg with salmonids (Melamed et al. 2004), humans (Yu et al. 2008), and sturgeon (Hale et al. 2010) have all subsequently been refuted (Wijayawardena et al. 2015). As rare transfer events can provide insights into the evolutionary history of organisms and their relatedness (Ivancevic et al. 2013; Daubin and Szöllősi 2016 ), we aimed to further characterize and confirm our initial observation of HTT of Perere-3 and Sr3 and examine evidence for the direction of transfer.

Our first focus was on assessing Perere-3 and Sr3 within the *S. mansoni* genome. Perere-3 and Sr3 are two LINE-RTEs with high sequence similarity that exist at high frequency in the *S. mansoni* genome. With a percentage identity of 79.8% across their full-length sequence, these elements fall just outside the “80:80:80 rule” (Wicker et al. 2007) which justifies their classification as separate TE families (Fig. 2a). We demonstrate that the diversity of near-full-length insertions of Perere-3 and Sr3 in the *S. mansoni* genome segregated into two distinct clusters (Fig. 2b). We propose that these elements were at one point a single element that underwent extensive transposition and divergence in *S. mansoni*.

Within the *S. mansoni* genome, Perere-3 and Sr3 represent the second and fourth most abundant TEs, respectively (Fig. 2c). Together, these elements comprise 6% of the *S. mansoni* genome and account for approximately one-fifth of the organism's TE content, indicating extensive past transposition. In addition to their considerable abundance, we show that these elements are capable of transposition, as indicated by 432 insertions having full-length (Fig. 2d) conserved L1-EN and RT protein domains, the two enzymes needed for active transposition. In fact, these TEs are currently, or were until very recently, actively transposing, as evidenced by the 430 copies with terminal branch lengths of 0 (Fig. 2e), ie not even one nucleotide difference between the pairs of insertions considered. Historical transposition bursts and periods of ongoing activity may suggest a lack or reduced capability of sequence-specific resistance by the host genome, making these TEs good candidates of HTT (Gilbert and Feschotte 2018). Perere-3 and Sr3's high transposition activity in *S. mansoni* has resulted in high TE abundance and concomitant increased genome size. In addition, it provides a large source of homologous substrate for recombination, which may have contributed in turn to the expansion of gene families in the TE-rich subtelomere of *S. mansoni* (Brann et al. 2024).

We further investigated the presence or absence of Perere-3 and Sr3 across *Schistosoma* and Panpulmonata (molluskan) lineages by manually curating consensus sequences of these TEs in selected species. We were able to identify and curate Perere-3 and Sr3-like elements from representative *Schistosoma* lineages having constructed a phylogeny for all genome assemblies available at the time of writing. We show that the most recently diverged *Schistosoma* species (*S. haematobium*, *S. spindale*, *S. mansoni*, and *S. rodhaini*) have a larger proportion of active elements (reflected in the lower TBL median) than the more basal species *S. japonicum* and *S. turkestanicum* (Fig. 3a). We cannot directly attribute speciation of *Schistosoma* to the horizontal acquisition of an element in the last common ancestor, but such an event would have had a large impact on the genus’ genome evolution, interrupting genes and/or genic regions or promoting structural rearrangements. Venancio et al. (2010) hypothesized that the transposition of Perere-3 and Sr3 contributed to the speciation of African schistosomes. To confirm this, “speciation genes” associated with *Schistosoma* diversification would first need to be identified (Nosil and Schluter 2011) and the subsequent contribution of Perere-3 and Sr3 to these be investigated.

Owing to Perere-3 and Sr3 very high sequence similarity (79.8%) in *S. mansoni*, we considered these TEs as functionally equivalent for assessing their presence in non-Schistosomatidae genomes. Our analysis identified significant hits of Perere-3/Sr3-like sequences in Planorbidae genomes, a family of snails susceptible to *Schistosoma* infection. The large evolutionary distance that separates these two animal groups, approximately 600 million years (Kumar et al. 2022), renders vertical inheritance of these highly similar sequences quite unlikely and may be better explained by horizontal transfer. To further investigate the extent of Perere-3/Sr3 presence in mollusks, we extended our relaxed BLASTN to include other, related snails from *Hygrophila* and the wider Panpulmonata (Fig. 3b). Given the multiple occurrences and completeness of copies found across molluskan genomes, such an observation is unlikely to result from DNA contamination during genome assembly, as has been proposed for previous putative *S. mansoni* HGT observations (Wijayawardena et al. 2015). Interestingly, *Bi. glabrata* and *Bi. pfeifferi* had only degraded copies, despite their close relationship to *Bi. straminea* (Fig. 3b), suggesting that in these genomes transposition stopped sometime post-divergence from *Bi. straminea*.

To find further evidence in support of HTT and discern potential evolutionary relationships between TE-derived sequences in *Schistosoma* and panpulmonate, we calculated the pairwise nucleotide distance of manually curated, consensus sequences of Perere-3/Sr3-like from species in these groups (Fig. 3c). We show that *Schistosoma* Perere-3/Sr3-like sequences cluster within the comparatively more diverse molluskan sequences. This observation suggests a potential route for the horizontal transfer of Perere-3/Sr3 from panpulmonate to *Schistosoma* presumably via the parasitic relationship of an ancestral *Schistosoma* panpulmonate host–parasite pair. The two groups of molluskan sequences formed by the “insertion” of the *Schistosoma* cluster (Fig. 3c) only loosely correspond to either the constructed phylogenetic tree or panpulmonates’ susceptibility to schistosome infection. This discordance could be explained by yet undescribed parasitic relationships between snail and host, or relationships that cannot occur due to a lack of geographic overlap rather than biological incompatibility. From the sequence and species relationships investigated here, we propose that Perere-3/Sr3 LINE-RTEs, which have extensively transposed in *Schistosoma* spp., could have been acquired via horizontal transfer from an ancestral snail host.

We generated further evidence in support of this hypothesis using synonymous substitution rates as a comparative framework. If Perere-3/Sr3-like elements had been vertically inherited since the divergence of mollusks and flatworms, their synonymous divergence would be expected to approach the levels observed among conserved orthologous genes across these phyla. Instead, the synonymous divergence observed between schistosome and snail RT pol sequences was markedly lower than that of BUSCO orthologs, many of which show evidence of saturation at this evolutionary depth. At the same time, RT pol sequences within Schistosoma exhibit levels of synonymous divergence comparable to those observed among vertically inherited orthologs between schistosome species, suggesting that once established in the schistosome lineage, these elements have evolved under similar substitution dynamics to host genes. The presence of very low dS values among some copies within *S. mansoni* further indicates recent duplication events and ongoing activity of these transposable elements. Together, these patterns are consistent with a scenario in which Perere-3/Sr3-like elements entered the schistosome lineage after the divergence of mollusks and flatworms and subsequently expanded within schistosome genomes, rather than being inherited vertically from a distant common ancestor.

To better understand the context of these HTT, we generated a phylogeny of all user-submitted LINE-RTEs from RepBase. This identified that, firstly the Perere-3/Sr3 sequences are distinct from other known transferred LINE-RTEs, such as BovB and AviRTE and, secondly, that several submitted sequences from unrelated, non-*Schistoma* and nonmolluskan, species cluster in and around the Perere-3/Sr3 sequences. To investigate if the presence and potential transfer of these TEs could extend to more distantly related species, we expanded our search through a collection of metazoan genomes. In this approach we queried the genomes of 2,271 organisms (for which speciation data were available at timetree.org and N50 was greater than 1Mbp) for sequences with high similarity to *S. mansoni* Perere-3 or Sr3. We identified Perere-3/Sr3-like sequences across 298 organisms, 204 of which were classified as “strong” hits (more than five hits larger than 1,000 bp and one more than 2,000 bp), including reptiles, gastropods, and fish (Fig. 4b). A weak hit was identified from one mammal (*Gracilanus agilis*) and bird (*Pseudopodoces humilis*). These weak hits likely represent contamination of the genome assembly, as the multiple large copies were not identified, indicating transposition had not occurred in these genomes. Other species had strong hits, indicative of not only the presence of a single TE but also TE activity. Of these, there may be a biological rationale for the presence of Perere-3/Sr3 in certain species, for example, the painted turtle *C. p. bellii*, which organisms from the superfamily *Schistosomatidae* do infect turtles, and trematode infection has been described (Johnson et al. 1998). Fish host a remarkable number of digenean parasites (Bullard and Overstreet 2008), and the horizontal transfer of genetic material between predator and prey has been documented via parasite intermediaries (Kambayashi et al. 2022). Fish, such as *Oreochromis* identified here have been trialed in the biological control of schistosomiasis via predation of snail populations (Slootweg et al. 1994) and will also eat free-living aquatic stages of the trematode life cycle (Orlofske et al. 2015). It is possible that the feeding of fish, on either Perere-3/Sr3-containing snails or schistosomes, may provide an avenue by which HGT could have occurred. Many of the identified snails are hosts for multiple parasites with complex life cycles; for example, *L. stagnalis* is “a cosmopolitan vector of trematodes infecting diverse vertebrates” (Gilbert et al. 2010). It is possible that these panpulmonates act as a distribution center for horizontally transferred material across different host–parasite combinations and life cycles that facilitate the transfer of Perere-3/Sr3 across metazoan organisms in and around aquatic environments. Water-filtering organisms such as sponges and corals accurately reflect the metagenome of a given environment (Cai et al. 2022). In aquatic environments where Perere-3/Sr3 are actively expanding within and transferring between organisms, it seems likely that this phenomenon would extend to organisms sampling such an environment.

The phylogenetic distribution of Perere-3/Sr3-containing species is patchy and unlikely to be the result of vertical inheritance alone, which would require many simultaneous TE losses to occur in many genomes. This observation suggests that the horizontal transfer of Perere-3/Sr3 extends well beyond the host–parasite relationships between *Schistosoma* and mollusks, which was further tested with a comparison of the speciation tree and the TE phylogeny generated for these organisms (Fig. 5), demonstrating phylogenetic incongruence. However, additional work will be required to robustly characterize any putative transfers that may have occurred beyond *Schistosoma* and its intermediate hosts. This should include formally contrasting molecular distances between orthologous, vertically inherited genes and Perere-3/Sr3-like elements across candidate taxa to better constrain the timing of putative transfers. Moreover, implementing appropriate clustering or phylogenetic frameworks to account for shared ancestral events will be essential for estimating the minimum number of horizontal transfer events needed to explain the wider metazoan distribution observed.

## Conclusion

We were able to leverage available genomic resources for *S. mansoni* and the high-quality genomes of other organisms to identify Perere-3 and Sr3-like sequences and characterize the extent of horizontal transfer of these LINE-RTEs in a way not previously possible. We show evidence in support of horizontal transfer of Perere-3/Sr3 from panpulmonate snails to *Schistosoma* parasites. This phenomenon may extend to other metazoan genomes, which demonstrated a patchy, discontinuous distribution with respect to the presence of Perere-3 and Sr3-like sequences, which, once constructed, also demonstrated phylogenetic incongruence. Owing to the rebuking of previous *S. mansoni* transfer events, we believe this represents one of the few robust horizontal transfer examples available in *S. mansoni*, due to the number of organisms implicated, quality of genomes sampled, and breadth of organisms involved.

## Methods

For a preliminary assessment of horizontal transfer in selected genomes (Table 3), we used a library of available and curated *S. mansoni* TEs (Table S4, File S1) as a query in a BLASTN search (v2.12.0) (Camacho et al. 2009) with predefined “relaxed” parameters that improve the identification of contiguous TE sequences in species where they may have diverged and/or be highly fragmented in relation to the query sequences used (Galbraith et al. 2020). These parameters are “-reward 3 -penalty -4 -xdrop_ungap 80 -xdrop_gap 130 -xdrop_gap_final 150 -word_size 14 -dust yes -gapopen 30 -gapextend 6 -outfmt 6 -evalue 1e-5” and define what we call, from now on, a “relaxed BLASTN.” In addition to published and available TEs (Drew and Brindley 1997; Drew et al. 1999; Laha et al. 2002; Arkhipova et al. 2003; Copeland et al. 2003; DeMarco et al. 2004 ; Feschotte 2004; Laha et al. 2004; Copeland et al. 2005; DeMarco et al. 2005; Laha et al. 2005; DeMarco et al. 2006; Jacinto et al. 2011), we manually curated Perere-3 and Sr3 consensuses (File S2) using the previously published sequences (DeMarco et al. 2005; Laha et al. 2005; Venancio et al. 2010) and published methodology (Goubert et al. 2022) with the *S. mansoni* genome (Buddenborg et al. 2021, PRJEA36577, WormBase ParaSite release WBPS19).

**Table 3:** Genome accessions used in preliminary identification of horizontally transferred elements

Organism	Assembly	Notes
*S. mansoni*	GCA_000237925.5	Parasite
*T. regenti*	GCA_944472135.2	Other Schistosomatidae
*H. americana*	GCA_944470555.2	
*F. hepatica*	GCA_948099385.1	
*C. sinensis*	GCA_003604175.2	Other trematodes
*Bu. truncatus*	GCA_021962125.1	Intermediate host
*Bi. straminea*	GCA_021533235.1	
*L. stagnalis*	GCA_900036025.1	
*H. sapiens*	GCF_000001405.40	Definitive

Organisms were selected for their relationship, either evolutionarily or parasitically to *S. mansoni*, as described in “Notes.”

We used the compiled TE library with manually curated Perere-3 and Sr3 to annotate the *S. mansoni* genome (Howe et al. 2017; Buddenborg et al. 2021) available in WormBase ParaSite release WBPS19 (PRJEA36577), using RepeatMasker v4.1.236 (Smit et al. 2013) with default parameters except for “-no_is -gff -s -a.” To investigate conserved protein domains in consensus sequences of Perere-3 and Sr3, we retrieved open reading frames with EMBOSS’ “getorf -minsize 500” (Rice et al. 2000) from their DNA sequences and submitted them to InterProScan5 (Jones et al. 2014). Percentage identities of consensus and relevant domains (L1-EN-cd09076 and RT-Pol-PS50878) were generated using Jalview's Pairwise Alignment (Waterhouse et al. 2009). When evaluating Perere-3/Sr3 genomic insertions, these were considered “complete” when conserved protein domains “Exo_endo_phos” (corresponding to L1-EN) and “RVT_1” (corresponding to RT Pol) were both present in at least 99% of their full length, as identified with Pfam (Mistry et al. 2021).

To study the relationship between Perere-3 and Sr3 insertions within the *S. mansoni* genome, we retrieved copies with a minimum length of 3,200 bp and used them to construct a distance tree with iqtree (v2.2.3) (Minh et al. 2020) with default parameters except for “–seqtype DNA -T AUTO –alrt 1000.” The resulting tree was visualized in ITOL (v5) (Letunic and Bork 2021) and Perere-3 and Sr3 colored to highlight segregation. We calculated relative TE quantities directly from our output of RepeatMasker (see above) and represented these using the “treemapify” (v2.5.5) and “ggplot2” (v3.4.3) (Wickham 2011) packages, implemented in R Studio (Build 554) (RStudio Team 2020). To investigate substitution rates of these elements with respect to their consensus sequences, we collected kimura values (with divCpGMod) from RepeatMasker's alignment file generated using the “-a” option and represented them in a histogram.

To further assess TE activity, we used the “terminal branch length” (TBL) method, previously used for analysis of zebrafish TEs (Chang et al. 2022). Fragments greater than 500 bp were aligned with “mafft —-auto” (Katoh et al. 2005), trimmed with TrimAl (Capella-Gutiérrez et al. 2009) with the “-gt” parameter set to 0.01. Trees were then constructed per TE using “FastTree -nt -gamma” (Price et al. 2010), and TBLs of the element fragment tree were then extracted using “termlength.py.”

To provide a framework for our proposed TE transfer among *Schistosoma* and selected species, we constructed a species tree using sequences obtained with reciprologs (https://github.com/glarue/reciprologs, with default parameters), an approach that identifies “reciprocal best hits,” ie amino acid sequences that are pairwise best hits across all species sampled. These reciprolog genes were then aligned per gene using “mafft —-auto” (Katoh et al. 2005) and subsequently concatenated to generate a single alignment per species of all reciprocal best hits. Species-level phylogeny was then constructed with iqtree (Minh et al. 2020) with parameters “–seqtype AA -T AUTO –alrt 1000 -B 1000.” For the input into reciprologs, we obtained trematode amino acid sequences from WormBase ParaSite (release WBPS19) and filtered them to include just the longest isoform of every gene. For the snail genomes, for which amino acid sequences were not readily available, BUSCO (Simão et al. 2015) was used (with default parameters and the “mollusca_obd10” database) to identify putative conserved amino acid sequences derived from the mollusk genomes. The resulting amino acid sequences were then used as inputs for reciprologs. Further to our identification of Perere-3/Sr3 sequences in non-*S. mansoni* genomes, we proceeded to manually curate (Goubert et al. 2022) their consensus sequences in these genomes (Tables 4, 5). We introduced a variation on the first step of the manual curation (see protocol by Goubert et al. 2022), replacing the stringent BLASTN search with our “relaxed BLASTN” approach to account for greater sequence differences between query (*S. mansoni* Perere-3 and Sr3) and subject.

**Table 4:** Genome accessions used in the construction of *Schistosoma* phylogeny

Organism	Assembly	Accession
*Plakobranchus ocellatus*	PoB_v1	GCA_019648995.1
*S. mansoni*	v10	GCA_000237925.5
*S. japonicum*	ASM636876v1	GCA_025215515.1
*S. turkestanicum*	tdSchTur1.1	GCA_944470395.2
*S. rodhaini*	tdSchRodh2.1	GCA_944470435.2
*S. spindale*	tdSchSpin1.1	GCA_946903255.1
*S. margrebowiei*	tdSchMarg1.1	GCA_944470205.2
*S. mattheei*	tdSchMatt1.1	GCA_944470405.2
*S. intercalatum*	tdSchInte2.1	GCA_944470385.2
*S. haematobium*	tdSchHaem2.1	GCA_944470465.2
*S. guineensis*	tdSchGuin1.1	GCA_944470375.2
*S. curassoni*	tdSchCurr1.1	GCA_944474815.3
*S. bovis*	tdSchBovi2.1	GCA_944470445.2
*Trichobilharzia regenti*	tdTriRege1.1	GCA_944472135.2

All genomes used are available at WormBase Parasite release 19. *Trichobilharzia regenti* was used as an outgroup for analysis.

**Table 5:** Genome accessions used in the construction of molluskan phylogeny

Organism	Assembly	Accession
*Plakobranchus ocellatus*	PoB_v1	GCA_019648995.1
*Achatina immaculata*	ASM976088v1	GCA_009760885.1
*Arion vulgaris*	ASM2079622v1	GCA_020796225.1
*Cepaea nemoralis*	Cnem_1.0	GCA_014155875.1
*Candidula unifasciata*	ASM976088v1	GCA_905116865.2
*Physella (Physa) acuta*	ASM2847654v2	GCA_028476545.2
*Lymnaea stagnalis*	v1.0	GCA_900036025.1
*Radix auricularia*	ASM207201v1	GCA_002072015.1
*Ampullaceana balthica*	Rbalt_genome_v1	GCA_944989445.1
*Bulinus truncatus*	Btru.v1	GCA_021962125.1
*Anisus vortex*	AniVort1.1	GCA_949126835.1
*Biomphalaria straminea*	Bstr_hk_v1	GCA_021533235.1
*Biomphalaria glabrata*	BioGlab47.1	GCF_947242115.1
*Biomphalaria pfeifferi*	UNM_Bpfe_1.0	GCA_030265305.1

All genomes used are available on the NCBI Genome List. *Plakobranchus ocellatus* was used as an outgroup for analysis.

To discard the possibility of contamination of snail genomes with *Schistosoma* DNA, we used the same “relaxed BLASTN” approach to assess similarity between the flanking 2,000 bp of each molluskan Perere-3 and Sr3 “hit” and the *S. mansoni* genome. Excluding *Anisus vortex*, no significant hits (>1,000 bp) were returned (note, as a control, using this same BLASTN approach with the Perere-3 and Sr3 could identify elements with >90% of full length). For *A. vortex*, 2,139 *S. mansoni* sequences were returned. These sequences had a minimum of 99.2% TE content and intersected with 2,211 *S. mansoni* Perere-3 and Sr3 sequences, indicating that these *A. vortex* flanking regions simply also contained Perere-3 and Sr3, for example, from several clustered elements. Placing a 99% TE-containing filter for molluskan flanking sequences returned no sequences from any molluskan organism tested (data not shown).

To investigate the relationship between consensus sequences of Perere-3 and Sr3-like elements from *Schistosoma* and snail genomes, we calculated their pairwise nucleotide distances (including that of Sr2 as outgroup) using EMBOSS’ “distmat” and the Jukes–Cantor substitution model (Rice et al. 2000). These relationships are represented using the “pheatmap” package (Kolde and Kolde 2015) to generate a hierarchical clustering tree.

To assess any potential transfer of Perere-3 and Sr3 across metazoan's TEs, we constructed a dendrogram of combined novel Perere-3/Sr3-like sequences from *Schistosoma* and molluskan organisms (Tables 4, 5) and LINE_RTE sequences from RepBase (Bao et al. 2015). Sequences were aligned with “mafft –auto” and trimmed with TrimAl (Capella-Gutiérrez et al. 2009) with “-gt” set to 0.25. This dendrogram was generated with iqtree using default parameters except for “-bb 1000 -alrt 1000.” To identify the extent of transfer of Perere-3 and Sr3 across a larger collection of metazoan genomes, we scanned 3,446 high-quality genome assemblies (scaffold N50 > 1Mbp, accessed from GenBank using the esearch function, August 2023) (Benson et al. 2018) using our “relaxed BLASTN” approach. The species list was filtered for entries with available speciation data (*n* = 2,271) at timetree.org (Kumar et al. 2022). We manually annotated the tree to indicate “taxa by phyla” for all nonchordates and “taxa by class” for chordates. Some clades representing a single or small numbers of species were not annotated to improve clarity of figure.

To test the horizontal gene transfer (HGT) hypothesis, we compared the synonymous divergence (dS) of TE and BUSCO orthologs across five species: two well-annotated schistosomes (*S. mansoni* and *S. japonicum*) and three freshwater snails (*Radix auricularia*, *Austropeplea immaculata*, and *Bi. straminea*). Reverse transcriptase (RT pol also called RVT) sequences belonging to the Sr3 family were retrieved from our database for all species. Only sequences with a minimum length of 700 bp were retained for downstream analyses. The final dataset used for dS estimation comprised 413 sequences: 69 from *S. japonicum*, 252 from *S. mansoni*, 50 from *Bi. straminea*, 27 from *A. immaculata*, and 15 from *R. auricularia* (Table S2). Nucleotide sequences were translated using Transeq (EMBOSS), and protein sequences were aligned with Clustal Omega (Sievers et al. 2011). The resulting alignment was back-translated to codon alignments using Tranalign. A phylogenetic tree was reconstructed with iqtree (Minh et al. 2020), using amino acid sequences as input and default parameters except for “-bb 1000 -nt 10 -m TEST.” Synonymous substitution rates (dS) were estimated across the phylogeny using the codeml program from the PAML v4.9j (Yang 2007) package, applying the branch model (model = 2, NSsites = 0). Pairwise cophenetic distances based on dS were calculated from the resulting tree using the cophenetic function in the ape package (Paradis and Schliep 2019) in R. In addition, a set of 205 single-copy orthologs was identified across the five species based on BUSCO annotations. dS values for these orthologs were estimated following the same pipeline described above for RVT sequences (Table S2).

To explore the species implicated in the metazoan transfer, we highlighted species with either the highest “max length hit,” “percentage identity,” or “number of hits over 1,000 bp.” We also included hits from notable taxa—the only bird, mammal and a coral (the highest of the seven corals identified), and *S. japonicum* (the only *Schistosoma* organism with speciation data). To explore horizontal transfer across these metazoan species, we first manually curated Perere-3/Sr3-like TEs for all organisms in Table 2 and generated both a species phylogeny and TE consensus tree for comparison. Species phylogeny for organisms in Table 2 was generated with timetree.org (Kumar et al. 2022), and TE consensus tree was constructed with iqtree and “-bb 1000 -wbt -alrt 1000” parameters after alignment with “mafft —-auto.”

## Supplementary Material

evag107_Supplementary_Data

## Data Availability

All processing, analysis, and visualization scripts used in this publication are available on GitHub at: https://github.com/tbrann99/Schisto_HTT/.
