# Supplementary material for: Host–Parasite Interactions Revisited: Evidence of Horizontal Transfer of a Transposable Element Between a Snail and Its Parasite
Source: Genome Biol Evol. 2026 May 8;18(5):evag107. doi: 10.1093/gbe/evag107 (PMC13155389; doi:10.1093/gbe/evag107)
Supplement: evag107_Supplementary_Data [file evag107_supplementary_data.zip › Supplementary Figure 1.pdf]

Supplementary Figure 1

Supplementary Figure 1 - Orthologous copies of Perere-3 / Sr3 suggest presence of TEs prior to *Schistosoma* speciation. Orthologous genes identified via previously described ‘reciprologs’ workflow. Groups of reciprologs were used as query for a blastn homology search using Perere-3 as subject. **A and B** - Gene models show location of the Perere-3 / Sr3 insertion in *S.mansoni* and *S.rodhaini* (*S.mansoni* clade) but absent from *S.haematobium* (*S.haematobium* clade) shown for reference. Tables below images show the blast results. The both examples, the insertion of the TE occurred in a common ancestor to *S.mansoni* and *S.rodhaini* after the split from the branch that led to *S.haematobium.* C - One Perere-3 / Sr3 insertion is vertically inherited in six species of the *S.haematobium* clade (*S. bovis, S. curassoni, S.guineensis, S.haematobium, S.intercalatum* and *S.maegrebowiei*), the shorter copy in *S. bovis, S. curassoni, S.guineensis* has presumably been truncated in a common ancestor to these three species but is still longer in *S.haematobium, S.intercalatum* and *S.maegrebowiei*)

A

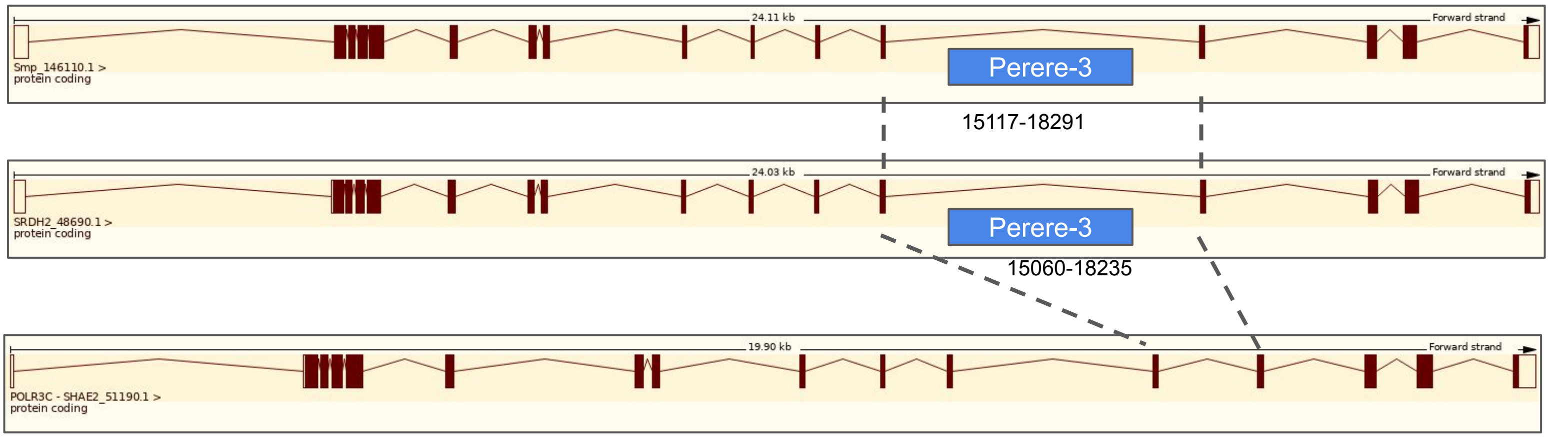

query	subject	%identity	length	mismatch	gapopen	query_start	query_end	subject_start	subject_end	evalue	bitscore
Smp_146110.1(+)	Perere-3	78.855	3197	627	42	15117	18291	3207	38	0	2113
SRDH2_48690.1(+)	Perere-3	78.924	3198	624	43	15060	18235	3207	38	0	2126

B

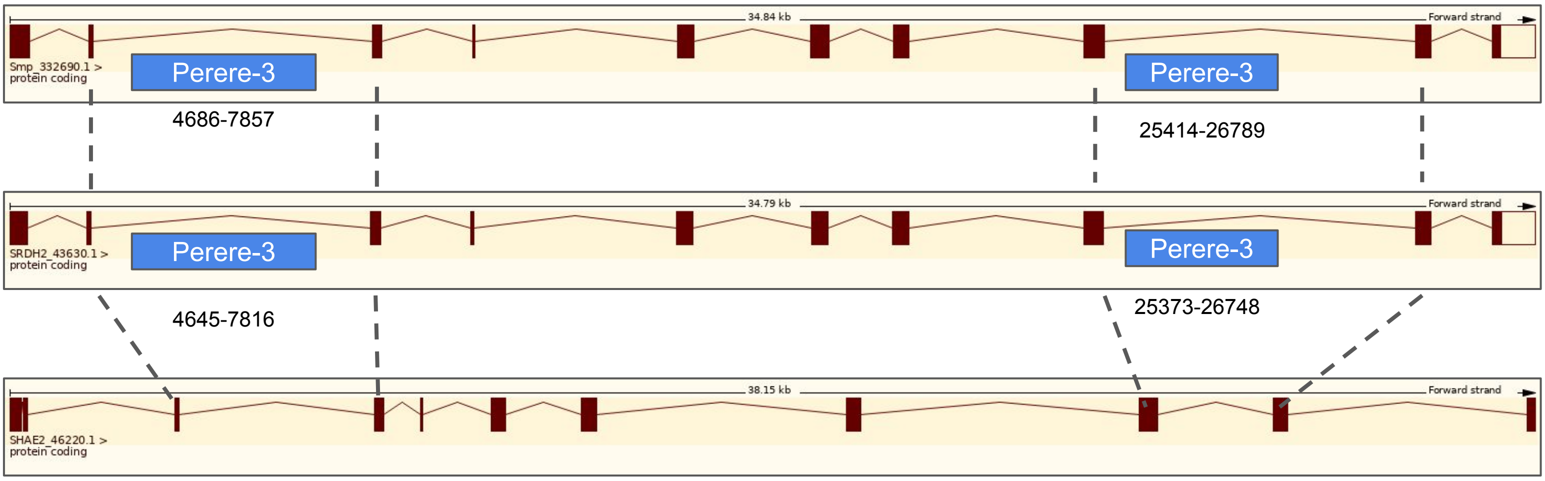

query	subject	%identity	length	mismatch	gapopen	query_start	query_end	subject_start	subject_end	evalue	bitscore
Smp_332690.1(+)	Perere-3	76.992	3225	655	60	4686	7857	3191	1	0	1764
Smp_332690.1(+)	Perere-3	87.616	1405	142	10	25414	26789	3195	1794	0	1602
SRDH2_43630.1(+)	Perere-3	77.223	3227	644	62	4645	7816	3191	1	0	1803
SRDH2_43630.1(+)	Perere-3	87.616	1405	142	10	25373	26748	3195	1794	0	1602

C

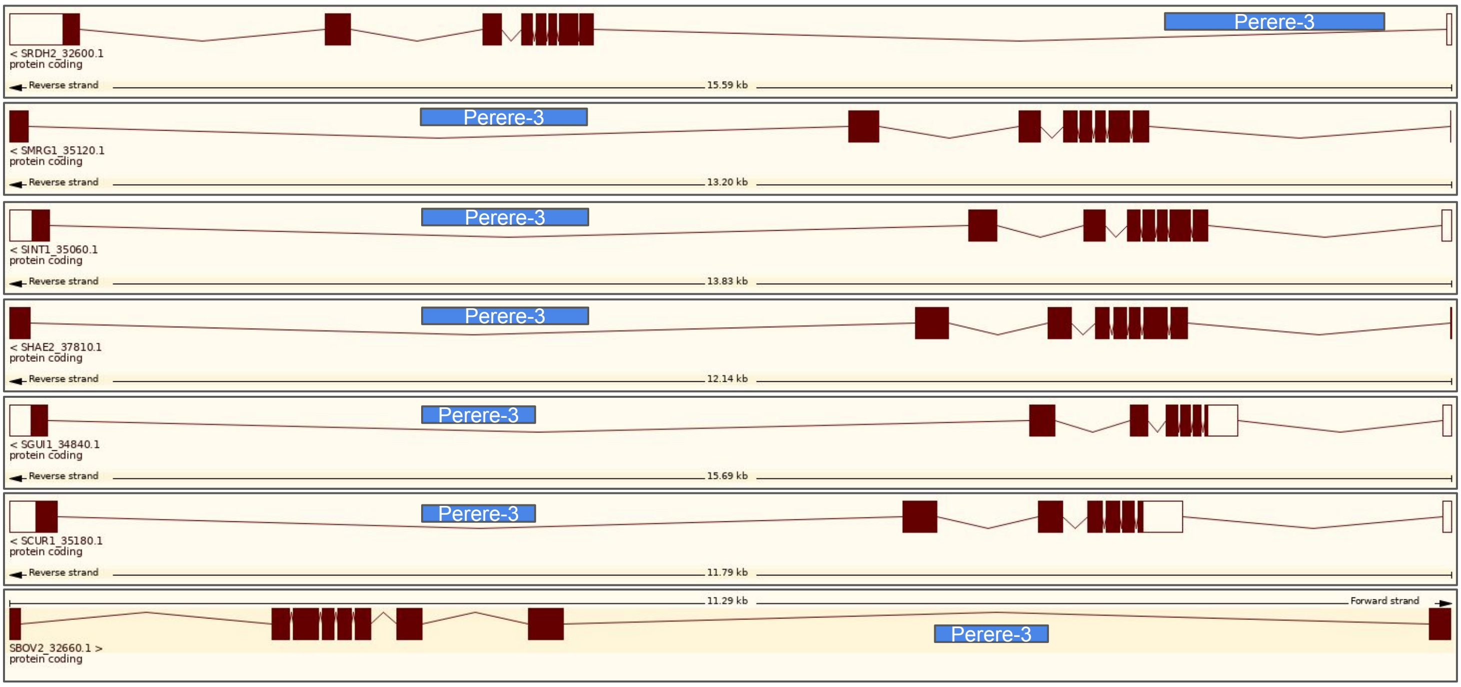

query	subject	%identity	length	mismatch	gapopen	query_start	query_end	subject_start	subject_end	evalue	bitscore
SRDH2_32600.1(-)	Perere-3	79.263	3231	611	54	2557	5759	3200	1	0	2200
SMRG1_35120.1(-)	Perere-3	83.916	2201	272	31	9738	11873	2191	8	0	2028
SINT1_35060.1(-)	Perere-3	85.351	2205	241	23	10156	12292	2191	1	0	2207
SHAE2_37810.1(-)	Perere-3	85.382	2018	247	16	8672	10642	2191	175	0	2049
SGUI1_34840.1(-)	Perere-3	84.025	1133	155	10	12853	13959	1307	175	0	1066
SCUR1_35180.1(-)	Perere-3	83.142	1133	153	15	8832	9926	1307	175	0	1000
SBOV2_32660.1(+)	Perere-3	84.113	1133	154	10	8693	9799	1307	175	0	1072
