# Supplementary material for: Host–Parasite Interactions Revisited: Evidence of Horizontal Transfer of a Transposable Element Between a Snail and Its Parasite
Source: Genome Biol Evol. 2026 May 8;18(5):evag107. doi: 10.1093/gbe/evag107 (PMC13155389; doi:10.1093/gbe/evag107)
Supplement: evag107_Supplementary_Data [file evag107_supplementary_data.zip › Supplementary Figure 2.pdf]

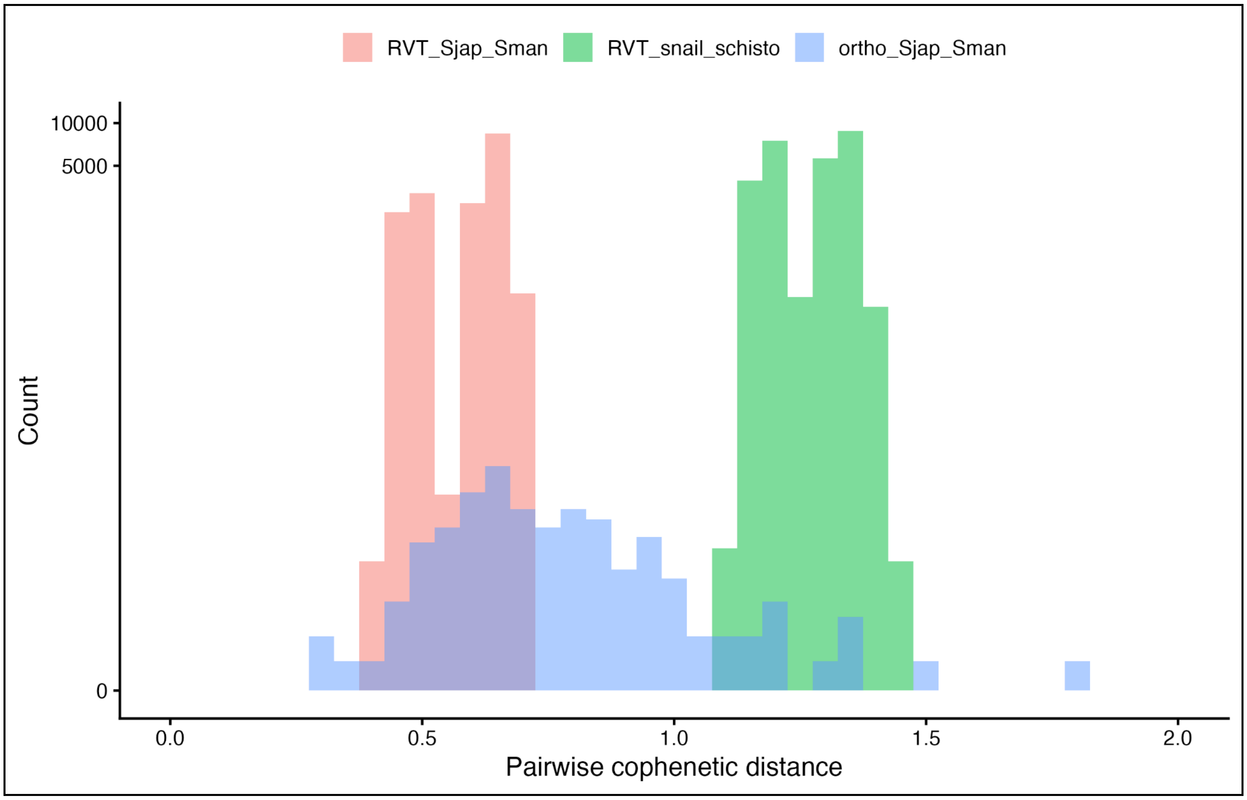

Supplementary Figure 2 - Distribution of synonymous divergence (dS) for TE-derived RVT sequences and conserved orthologs. Overlapping histograms show pairwise dS estimates for Sr3-family reverse transcriptase (RVT) sequences and 205 BUSCO single-copy orthologs across S. mansoni, S. japonicum, and three freshwater snails (Radix auricularia, Austropeplea immaculata, Biomphalaria straminea). RVT comparisons include intra- and inter schistosoma, and schistosome–snail pairs. Only dS values between 0 and 2 are shown, pairwise cophenetic values between snails and schistosoma are excluded.
