# Supplementary material for: Host–Parasite Interactions Revisited: Evidence of Horizontal Transfer of a Transposable Element Between a Snail and Its Parasite
Source: Genome Biol Evol. 2026 May 8;18(5):evag107. doi: 10.1093/gbe/evag107 (PMC13155389; doi:10.1093/gbe/evag107)
Supplement: evag107_Supplementary_Data [file evag107_supplementary_data.zip › Supplementary Figure 3.pdf]

Supplementary Figure 3

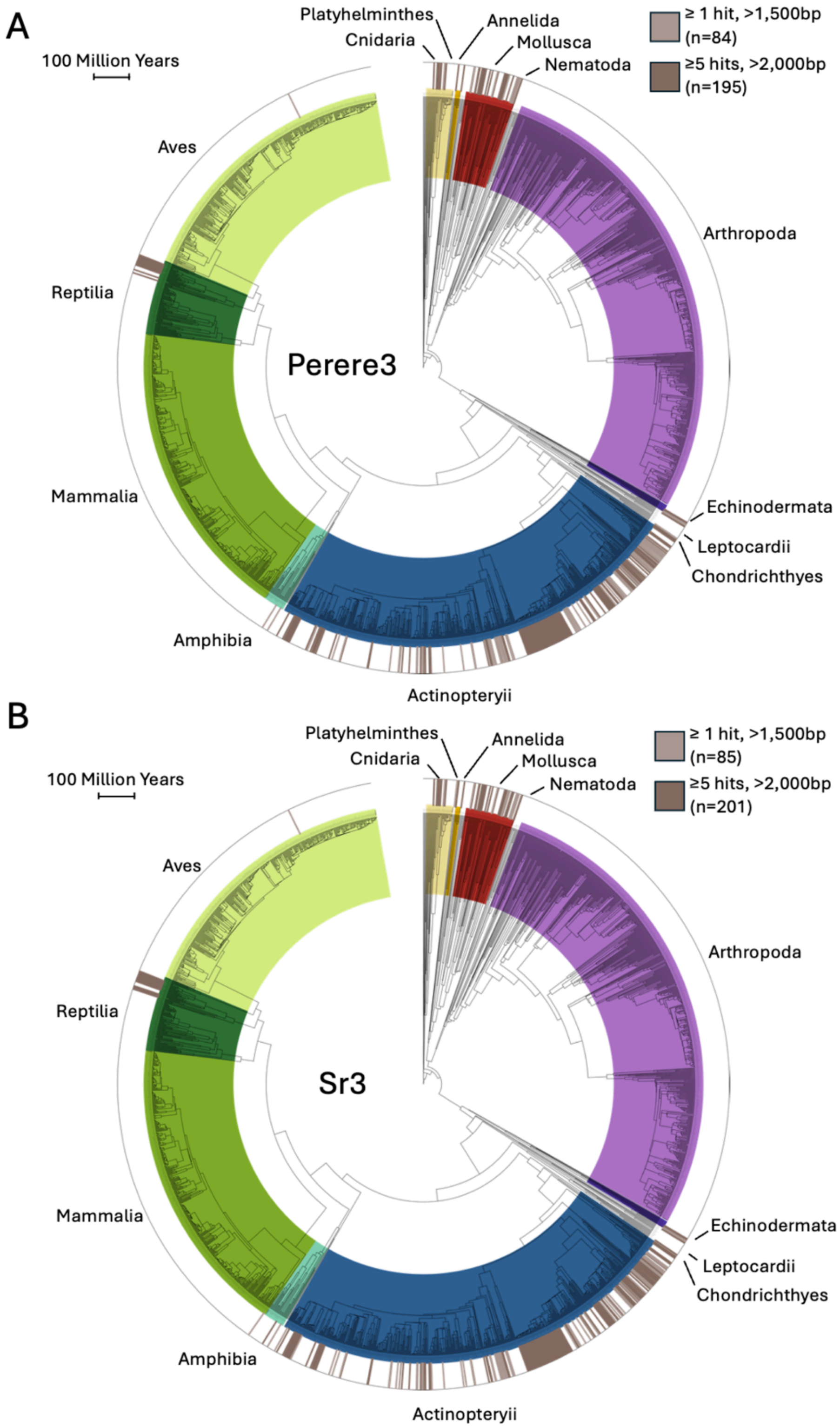

Supplementary Figure 3 - Separate Perere-3 (A) and Sr3 (B) blast results across high-quality metazoan genomes. Presence of Perere-3 / Sr3-like sequences across metazoan genomes available at Timetree.org. Relevant hits were classified as either weak (light brown: at least one hit > 1,500bp) or strong (dark brown: at least one hit > 2,000bp and at least five hits > 1,000bp).
