# Supplementary material for: Host–Parasite Interactions Revisited: Evidence of Horizontal Transfer of a Transposable Element Between a Snail and Its Parasite
Source: Genome Biol Evol. 2026 May 8;18(5):evag107. doi: 10.1093/gbe/evag107 (PMC13155389; doi:10.1093/gbe/evag107)
Supplement: evag107_Supplementary_Data [file evag107_supplementary_data.zip › Supplementary Figure 4.pdf]

Supplementary Figure 4

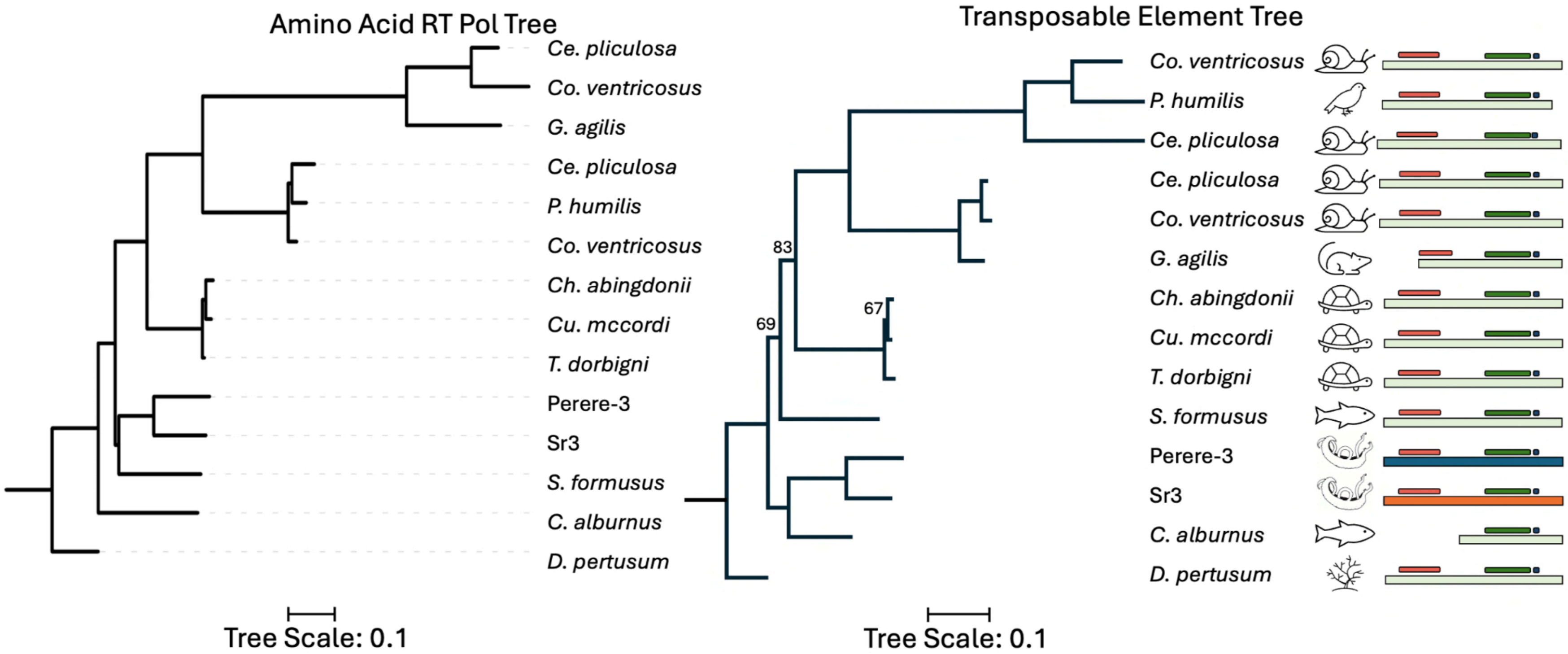

Supplementary Figure 4 – Reverse transcriptase and transposable element consensus distance trees provide similar topology. Trees of manually curated TE consensus sequences of Perere-3 and Sr3 from organisms of Table 4. Two distinct elements were curated for *Conus ventricosus* and *Cerithideopsis pliculosa*. *Culter alburnus* and *G. agilis* curated elements were 5' truncated. Reverse transcriptase domain tree generated using InterProScan5 to identify relevant protein domains. Both trees generated using '`iqtree -bb 1000 -wbt -alrt 1000`' after alignment with mafft.
